# Exploring the Structure–Activity Relationship of Bentonites for Enhanced Refinement of Recycled Vegetable Oil

**DOI:** 10.3390/ma18051059

**Published:** 2025-02-27

**Authors:** Alberto Mannu, Simona Castia, Giacomo Luigi Petretto, Sebastiano Garroni, Franca Castiglione, Andrea Mele

**Affiliations:** 1 Department of Chemistry, Materials and Chemical Engineering “G. Natta”, Politecnico di Milano, Piazza L. da Vinci 32, 20133 Milano, Italy; franca.castiglione@polimi.it (F.C.); andrea.mele@polimi.it (A.M.); 2 Department of Chemical, Physics, Mathematics and Natural Science, INSTM, University of Sassari, Via Vienna 2, 07100 Sassari, Italy; s.castia@studenti.uniss.it (S.C.); sgarroni@uniss.it (S.G.); 3 Department of Medicine, Surgery and Farmacy, University of Sassari, 07100 Sassari, Italy; gpetretto@uniss.it

**Keywords:** solid-state NMR, bentonite, vegetable oil, pour point, design of experiments

## Abstract

The use of bentonite for recycling vegetable oils presents challenges, as even minor variations in the clay composition and structure can lead to significant differences in its ability to retain various chemical groups. This study investigates the structure–activity relationship of four bentonites—two hydrophilic and two hydrophobic (both in commercial and ground forms)—to better understand these effects. Solid-state NMR spectroscopy revealed subtle differences between hydrophobic and hydrophilic materials, as well as distinctions between ground and unground hydrophilic clays, through ^29^Si and ^27^Al experiments. These structural variations directly influenced the bentonites’ capacity to retain specific chemical groups, which in turn affected the pour point and volatile profile of the processed oils. A simplex lattice design of experiments, combined with multivariate analysis, facilitated the development of a predictive model to optimize process efficiency. Remarkably, this model achieved an improvement in pour point of up to 14.5 °C (from −2 °C to −16.5 °C) for oils treated with hydrophilic unground bentonite. This research underscores the critical role of bentonite morphology in enhancing the efficiency of vegetable oil recycling.

## 1. Introduction

Recycling waste cooking oils (WCOs) have emerged as an important topic in recent years [1,2]. Within the available methods and technologies for WCO refining for non-biodiesel applications, water treatment under controlled conditions has proven able to provide a high-purity recycled vegetable oil [3,4]. Nevertheless, while the purity assessment conducted through ^1^H NMR spectroscopy indicates a high level of triglyceride purity [5] upon water processing, minor constituents remain detectable, as revealed by UV-VIS [6] or SPME-GC [7] analyses. These impurities arise from several sources and are associated with the lifecycle of the oil prior to recycling.

WCOs can be contaminated by metal traces, aldehydes, ketones, heterocycles and other organic molecules originating from cooking tools, the Maillard process [8,9] and food leaching [10]. These minor components can also be removed by treatment with adsorbent materials such as natural clays or alternative powders [11]. Preliminary analysis of the volatile fraction prior to and after bentonite treatment of WCO samples revealed a complex mechanism underlying the action of bentonite. The results indicated that even slight variations in clay composition led to the preferential removal of different chemical groups [12]. This structure–activity relationship can also be observed by UV-VIS spectroscopy [6].

In this context, any knowledge regarding the mechanisms behind the absorption capacity of clay can contribute to the development of specific protocols aimed at vegetable oil recycling processes. Recently, a study has been conducted on the recycling of used vegetable oils with hydrophilic or hydrophobic bentonites, revealing the pivotal role of hydrophobicity in the removal of polar compounds from waste [13]. In that study, the presence of a small amount of water was crucial in determining the effectiveness of hydrophobic bentonites when used with recycled vegetables oils. Looking at recent studies, the topic of bentonite processing of WCOs has been thoroughly addressed [14,15]. Nevertheless, recent sustainability estimations [5] have indicated that water processing is more feasible than clay adsorption. In this context, two relevant aspects were not addressed: the use of bentonite clays to treat already recycled used vegetable oils, and the reason for the different performances within hydrophilic and hydrophobic bentonites sold with the same CAS number and apparently with the same physical characteristics.

Thus, four bentonites were used to capture the volatiles present in a recycled mixture of triglycerides, considering the variation in the pour point of the treated oil, which served as an indicator to highlight different outcomes related to different bentonites. Using a design of experiments (DOE) approach, the potential to prepare mixtures of different bentonites to be exploited as hybrid media in vegetable oil recycling has been assessed. A detailed characterization of the bentonites was carried out by solid-state magic angle spinning (MAS) NMR (^29^Si and ^27^Al), providing valuable insights into the morphology of the materials in relation to their properties, allowing us to relate the coordination mode of Si and Al atoms with the physical properties of the material.

## 2. Materials and Methods

### 2.1. Chemicals

Hydrophilic S001 (MKCR3493) and hydrophobic S002 (MKCN7873) bentonites were purchased from Merk Europe, Darmstadt, Germany (CAS number 1302-78-9).

### 2.2. Ball Milling

A total of 8 g of bentonite (S001 or S002) was ball-milled for 20 min in an SPEX 8000 M MIXER/MILL (Metuchen, NJ, USA) shaker mill at 875 rpm. The as-obtained S001M1 and S002M1 samples were then used for oil refinement.

### 2.3. Vegetable Oil Processing

For vegetable oil processing, 100 g of vegetable oil and 15 g of clay (15 wt%) were placed in a beaker, and the mixture was stirred for 30 min and then filtered under vacuum. The processed oil was then subjected to pour point analysis.

### 2.4. Pour Point Determination

The ASTM D97 method was adapted to our purposes and employed for pour point determination [16]. Oil samples (20 g) were placed in a falcon and slowly cooled in an ice/salt bath. The temperature of the oil was monitored by an internal thermometer every minute, until visual confirmation of the change from liquid to solid.

### 2.5. Headspace Solid-Phase Microextraction (HS-SPME)

A 100 μm PDMS/DVB/CAR (Polydimethylsiloxane/Divinylbenzene/Carboxen) coated fiber 50/30 Stableflex (Supelco, Sigma Aldrich, St. Louis, MO, USA) was preconditioned at 270 °C for 1 h. A total of 5 g of sample was added to a 20 mL SPME vial (75.5 × 22.5 mm) equipped with a septum. The system was conditioned for 5 min at 60 °C. Thus, the fiber was exposed to the headspace for 30 min. Once the extraction was completed, the fiber was desorbed for 2 min into the injector at a temperature of 250 °C in a splitless injection mode.

### 2.6. Gas Chromatography–Mass Spectrometry (GC-MS) Analysis

GC-MS measurements were carried out using an Agilent 6890 GC (Palo Alto, CA, USA), coupled with an Agilent 5973 MSD detector (Palo Alto, CA, USA). A ZB-Wax 30 m × 0.25 mm i.d., 0.25 µm film thickness column was used for the chromatographic separation. The following temperature ramp was used: held at 40 °C for 4 min, increased to 150 °C at a rate of 3.0 °C/min, held for 3 min and then increased to 220 °C at a rate of 10 °C/min. Finally, the temperature was held for 10 min. The constant flow of helium (carrier gas) was at a rate of 1 mL/min. The data were analyzed using a MassHunter Workstation B.06.00 SP1 (Agilent, Palo Alto, CA, USA). Identification of the analytes (Section 3.3) was achieved by comparing them with co-injected standards and matching their MS fragmentation patterns and retention indexes against built-in libraries, literature data or commercial mass spectral libraries (NIST/EPA/NIH 2008; HP1607 from Agilent Technologies, Palo Alto, CA, USA).

#### Retention Indexes

A hydrocarbon mixture of n-alkanes (C9–C22) was analyzed separately under the same chromatographic conditions to calculate the retention indexes with the generalized equation by Van del Dool and Kartz [17]:

Ix = 100[(t_x_ − t_n_)/(t_n+1_ − t_n_) + n] (1)

where t is the retention time, x is the analyte, n is the number of carbons of alkane that elute before the analyte and n + 1 is the number of carbons of alkane that elute after the analyte.

### 2.7. NMR Analysis

Solid-state NMR spectra were acquired with a Bruker NEO 500 spectrometer, equipped with a MAS iprobe, operating at 500.13 MHz for ^1^H, 99.35 MHz for ^29^Si and 130.31 MHz for ^27^Al. Solid samples were packed in a 4 mm zirconium oxide rotor. ^29^Si direct excitation (DE-MAS) spectra were acquired with a recycle delay of 20 s, spinning speed of 10 kHz and 560 scans. ^27^Al spectra were obtained with recycle delay of 5 s, spinning speed of 12 kHz and 256 scans. All the experiments were performed at 298 K. Deconvolutions were performed with origin 2024 using a Lorentzian fitting model. The chemical shifts are reported relative to tetramethylsilane (TMS) for ^29^Si using octakis-(trimethylsiloxy)silsesquioxane (Q8M8) and for ^27^Al using Al(NO_3_)_3_·9H_2_O as a secondary standard, respectively [18].

### 2.8. Karl Fischer Titration

Water content was determined through Karl Fischer titration with a coulometric MKV-710B titrator (Alkimia SRL, Milan, Italy).

### 2.9. FT-IR Spectroscopy

For the FT-IR measurements, a FT-IR Jasco 480 Plus spectrophotometer (Jasco International Co., Ltd., Tokyo, Japan) was employed. The powders were analyzed in the form of tablets prepared by mixing the bentonite with anhydrous KBr and pressing them into disks using a hydraulic press.

### 2.10. Design of Experiments (DoE)

A simplex lattice design was used to study the effects of four components in four runs. The design was run in a single block and the order of the experiments was randomized to minimize the effects of lurking variables. Statgraphics 18 software was used for the statistical multivariate analysis.

## 3. Results and Discussion

### 3.1. FT-IR Spectroscopy Analysis

**Figure 1:**
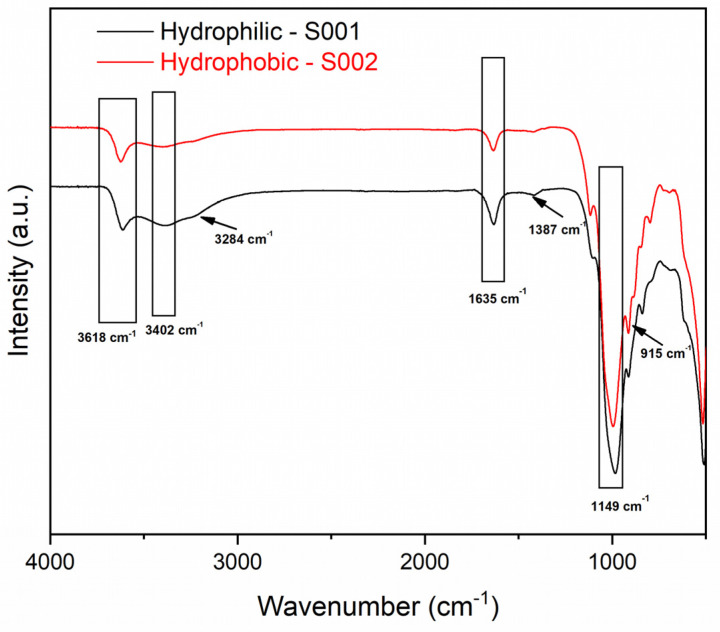
FT-IR spectra of hydrophilic S001 (black line, lower spectrum) and hydrophobic S002 (red line, upper spectrum) bentonites.

Bentonite is a natural material containing different minerals which can influence its properties [19]. According to the provider, the commercial bentonites herein considered are composed of montmorillonite, Al_2_O_3_ × 4SiO_2_ × H_2_O (molecular weight: 180.1 g/mol), with a particle size lower than 74 μm and a mean particle size of 5.45 μm [20]. Nevertheless, no additional data are available regarding the differences between the two products. Previous SEM and TEM analyses on the same batches of bentonites revealed, respectively, different interlayer distances and particle shapes, while powder X-ray analysis did not highlight any relevant difference [5]. Nevertheless, the behavior of the two clays, prior to and after grinding, was different, confirming the different properties of the apparently very similar materials. After being subjected to a ball-milling process, significant differences, in terms of microstructure, were observed, and interlayer distances were modified with respect to the pristine materials, probably due to the partial release of trapped water upon grinding [13]. To detect variations in the chemical groups, FT-IR analysis was conducted on both bentonites (Figure 1).

Looking at the spectra shown in Figure 1, it is possible to clearly see the different IR profiles of S001 and S002 bentonites. It is known that hydrophobic bentonites are obtained by chemical treatment of hydrophilic ones with the consequent removal of minor components, resulting in a less complex mixture of chemical groups in the FT-IR spectrum [21]. Regarding the specific composition observed, both samples show typical bands relative to the Si-O stretching (1149 cm^−1^), the bending of the adsorbed water (1635 cm^−1^) and the stretching of the adsorbed water (3402 cm^−1^), as well as the OH-Al stretching band (3618 cm^−1^). For the hydrophilic S001 bentonite, relevant bands associated with the -OH vibrations were detected at 3284 cm^−1^, and CO_3_ stretching of calcite was detected at 1387 cm^−1^. The band at 915 cm^−1^, visible in the hydrophobic S002 sample, can be attributed to Al-Al-OH bending [21].

**Figure 2:**
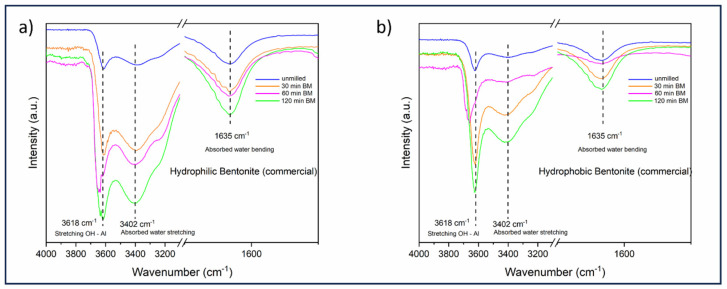
FT-IR spectra of hydrophilic (**a**) and hydrophobic (**b**) bentonite subjected to a ball-milling process (0, 30, 60 and 120 min).

In addition to the characterization of the starting materials, both the bentonites were monitored during the ball-milling procedure, specifically at 30, 60 and 120 min (Figure 2).

As already reported for similar samples subjected to the same milling treatment [13], it is possible to notice, for both bentonites, some slight changes in the FT-IR spectra after 60 min of ball-milling (shifting of the stretching OH-Al band and consistent attenuation of the adsorbed water bending band), followed by a further change, which apparently results from the restoration of the original chemical structure at 120 min. For this reason, the milled bentonites, which were further employed to treat the triglyceride mixture, were ground for 60 min.

### 3.2. NMR Morphological Analysis

To overcome the difficulties that occurred with the powder X-ray diffraction characterization and to add further information to the FT-IR spectroscopy data, thus providing a satisfying description of the differences between the bentonites studied, multinuclear solid-state NMR spectroscopy was performed. In fact, the limited selectivity of X-ray diffraction, commonly used for the characterization of such minerals, due to the tiny changes in scattering factor values can be overcome by looking at the solid-state NMR profile of the main heteroatoms [22]. In fact, Si and Al exhibit different gyromagnetic ratios γ (6.976 × 10^7^ rad T^−1^ s^−1^ for ^27^Al, and −5.3188 × 10^7^ rad T^−1^ s^−1^γ for ^29^Si), allowing us to highlight even small differences in the chemical environment of the analyzed nuclei.

**Figure 3:**
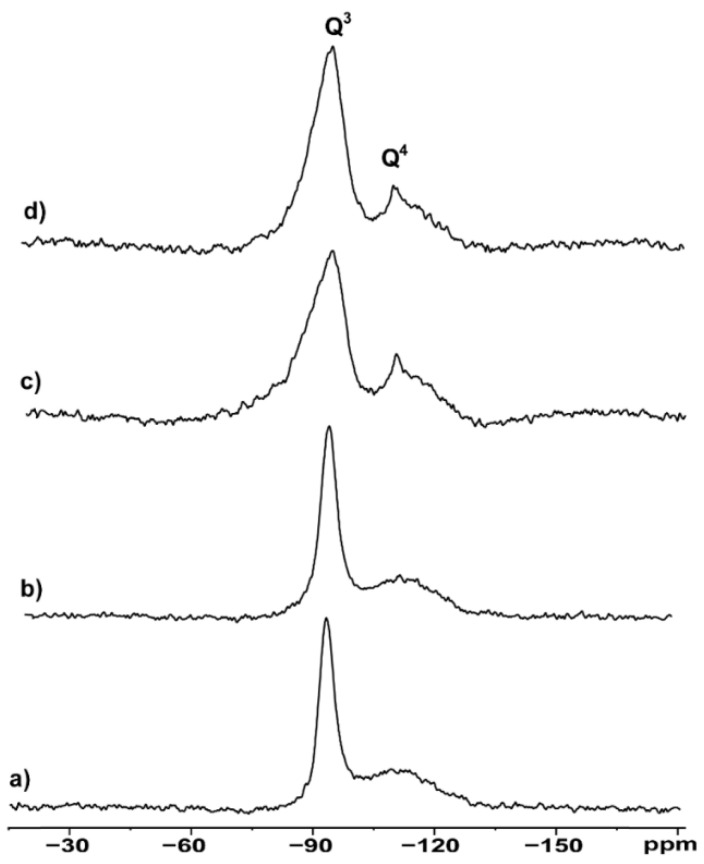
^29^Si MAS NMR spectra of (**a**) hydrophilic bentonite S001, (**b**) ball-milled hydrophilic S001M1, (**c**) hydrophobic bentonite S002 and (**d**) ball-milled hydrophobic bentonite S002M1.

Thus, ^29^Si MAS NMR analysis was performed on hydrophilic and hydrophobic bentonites as received and after grinding (Figure 3).

All spectra show a sharp peak at −93.4 ppm and a rather broad signal covering the range of −105–115 ppm, indicating local disorder of the Si environment in the 3D structure. According to the classic nomenclature and peak assignment proposed by Lippmaa et al. [23], the ^29^Si SS NMR signals in Figure 3 are labeled as Q3 and Q4, respectively. Q3 refers to “chain branching sites”, Si(OAl)(OSi)_2_OH in our case, whereas Q4 is associated with the “three-dimensional cross-linked framework” of the fully condensed Si(OAl)(OSi)_3_ frame, usually present in the tetrahedral layers of the clay [24]. After deconvolution of the spectral region containing the Q3 and Q4 signals, the peak’s width, as well as the absolute area ratios (Q4/Q3), were estimated and are reported in Table 1.

Hydrophilic bentonite shows a sharper Q3 signal compared to the hydrophobic one (491 Hz vs. 834 Hz) and a lower Q4/Q3 ratio (0.137 vs. 0.190). The higher Q4/Q3 ratio observed in the hydrophobic samples compared to their hydrophilic counterparts can be related to their lower Brönsted acidity compared to the hydrophilic samples. The rationale behind this is that Q3 contains the contribution of the Si-OH, which are responsible for the acidity. The effect of the ball-milling procedure is evaluated by comparing the suitable spectra in Figure 3a vs. Figure 3b for hydrophilic bentonites, and Figure 3c vs. Figure 3d for hydrophobic bentonites. The data highlight that grinding does not affect the chemical structure—as no chemical shift changes were observed—but rather it increases, in a selective manner, the structural disorder of the materials, as pointed out by the linewidth (LW) values. The variation, appreciable in Q4 especially, can be expressed as D_LW_ (Q4) = LW(Q4)_milled_ − LW(Q4)_not milled_ for both the hydrophilic and hydrophobic materials. The experimental data lead to values of D_LW_ (Q4)_hydrophilic_ = 9 Hz and D_LW_ (Q4)_hydrophobic_ = 55 Hz, providing evidence of more effective morphological changes in hydrophobic samples upon mechanical stress.

**Figure 4:**
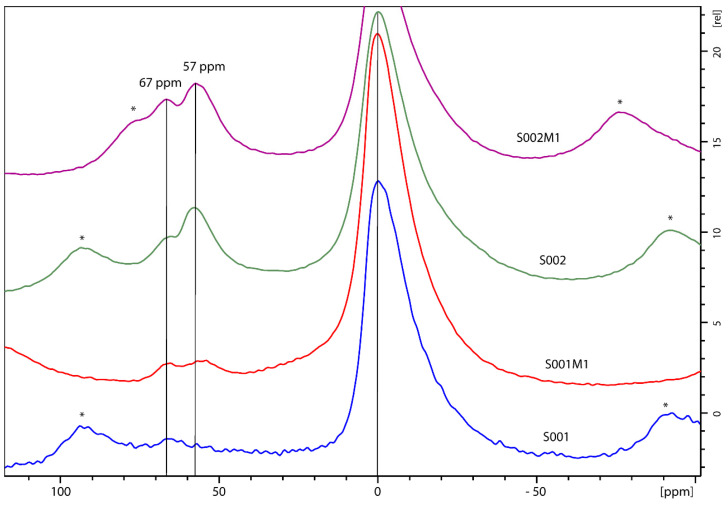
^27^Al MAS NMR spectra of hydrophilic bentonite S001, ball-milled hydrophilic bentonite S001M1, hydrophobic bentonite S002 and ball-milled hydrophobic bentonite S002M1. Spinning sidebands are marked with an *.

To provide an exhaustive overview of the chemical structure of the clays, ^27^Al MAS spectra were also acquired (Figure 4).

The experimental spectra show the presence of peaks in two regions, from 70 to 50 ppm and from −25 to +25 ppm, corresponding, respectively, to Al in its tetrahedral (Al^IV^) and octahedral (Al^VIII^) forms (Figure 4 and Table 2) [25]. It is known that the presence of Al in tetrahedral frameworks increases the Brönsted acidity of the material [26]. Also, two different peaks in the Al tetrahedral region can be observed, which correspond, respectively, to Al sites in layered bentonite (peak at around 70 ppm) and to the Al characteristics of fully condensed sites (55–59 ppm) [27]. The relative percentages of the areas of each peak are reported, along with the corresponding chemical shifts, in Table 2.

Looking at the data relative to the four bentonite samples analyzed, it is possible to draw the following conclusions. Hydrophilic bentonite (commercial) just shows octahedral Al sites (>91%) with about 8% tetrahedral Al layered sites. After ball-milling, about half of the layered sites are converted into fully condensed ones. The chemical shift, in this case for the fully compacted sites (about 56 ppm), is slightly anomalous as it should be close to 70 ppm. Thus, it cannot be excluded that this new peak corresponds to layered sites (not fully condensed) arranged in a different way than the original ones. However, a clear effect, concentrated on the tetrahedral Al sites, of the milling procedure is observed for hydrophilic bentonite. On the other hand, hydrophobic bentonites show all three configurations, with a distribution between octahedral, tetrahedral layered and tetrahedral fully condensed figurations, which seems to not be affected by the ball-milling procedure. Independently of the milling procedure, it is possible to understand the different behavior of the two types of bentonites (hydrophilic and hydrophobic) by considering their octahedral/tetrahedral site ratios, which are, respectively, about 92/8 and 75/25. As reported by Vajglova and coworkers [25], the presence of Al in the tetrahedral frameworks increases the Brönsted acidity of the material; thus, the bentonite sold under the label hydrophobic differs from the hydrophilic one as it has a consistent increased number of Bronsted acidic sites.

### 3.3. Design of Experiments (DoE)

Once the relevant structural differences between the considered bentonites were highlighted by solid-state NMR spectroscopy, a simplex lattice design of experiments (DoE) [26,27,28,29] was used to build a viable statistical model describing the effect of the specific bentonite used on the pour point of the processed oil. According to the DoE, four experiments were conducted, and for each one, the volatile content (VC) and pour point (PP) were determined (Table 3).

The volatile content was determined by SPME GC-MS analysis, which provided a semiquantitative assessment of several chemicals in different relative amounts (Table 4).

The general chemical compositions of the volatile fraction of the oil prior to and after treatment with bentonite are in agreement with previously reported HS-SPME GC/MS data relative to similar matrixes [7,30]. Looking at the main organic volatile compounds (VOCs) detected and reported in Table 4, it is possible to explain the presence of many organic compounds, such as aldehydes and acids, as resulting from food leaching [31], the Maillard reaction, oxidation promoted by temperature and the hydrolysis of triglycerides [32]. Furthermore, regarding the aldehyde profiles, the autoxidation of linoleic acid has been reported as a source of dienals (2,4-heptadienal or 2,4-decadienal) and alkenals (2-undecenal, 2-decenal and 2-octenal) [33].

To better compare the outcomes of the different treatments in terms of variations in their volatile profiles, the detected compounds have been grouped into chemical families, as shown in Table 5, which also integrates the PP values of each sample.

Looking at the data reported in Table 5, the most evident difference between the starting oil, TQ, and the treated ones is related to the PP, which consistently improves from −2 °C to about −10 °C for samples treated with bentonites S002, S002M1 and S001M1, reaching −16.5 °C after processing with bentonite S001. The 6.5 °C drop in the treated samples of hydrophobic bentonite (S002 series) compared to S001 is indicative of the specific ability of the hydrophilic bentonite to remove some chemicals. It is known that water molecules in waste vegetable oil samples are better removed by hydrophilic bentonites, and increasing the specific surface area can enhance this phenomenon [12]. Nevertheless, herein, we observe an inverse trend, as the non-milled hydrophilic bentonite is much more efficient that the milled one (S001 vs. S001M1). The latter conclusion seems to contrast with the published data [4]; regardless, it should be considered that when recycled vegetable oil is treated, a low tenor of water (0.4 wt% from Karl Fischer titration) is present than in raw used vegetable oil. In such an environment, the action of the bentonite is related to the trapping of organic molecules that have not been saturated by water, as happens in waste cooking oils [5]. It is also known that the absorption process of porous materials involves a combination of pore inclusion (determined by pore size) and the interactions between polar/non-polar groups. Regarding the differences in morphology, the ^29^Si MAS NMR spectra showed sharper Q3 signals in hydrophilic bentonites than hydrophobic ones, meaning that hydropilic bentonites show less contribution of Si(OAl)(OSi)_2_OH phases in their structures. Also, the enlargement of the peaks in the milled samples suggests a decrease in crystallinity due to the ball-milling process. On the other hand, the ^27^Al MAS spectra revealed that milled bentonites have a high-density packed structure (increasing the Al^IV^ peak). Thus, it seems that the combination between the reduced number of O-H groups and the more tiny and regular structure are responsible for the better performances of S001 than the other clays in removing organic contaminants.

Looking at Table 2, it is possible to gain some insights about the specific chemical groups retained during the four processes. By comparing columns 1 and 2 (hydrophilic bentonites) with 3 and 4 (hydrophobic bentonites), a reduction in the relative amounts of ketones, acids, alcohols and alkanes is observed after processing with hydrophobic bentonites. Considering that hydrophobic bentonites have fewer -OH groups, it is possible to attribute this behavior to the physical retention of the low-molecular-weight contaminants.

Looking for differences between milled and non-milled hydrophilic bentonites, the most relevant difference lies in the relative amounts of ketones and alcohols, which are reduced in S001-treated samples (non-milled). It is possible to relate this outcome to the reduction in Al^IV^ signal intensity in S001-processed oils (^27^Al MAS spectra). Again, there is an indication that the structural organization of the clay has a relevant impact on specific chemical groups’ retention ability.

### 3.4. Optimization by Statistical Multivariate Analysis

**Figure 5:**
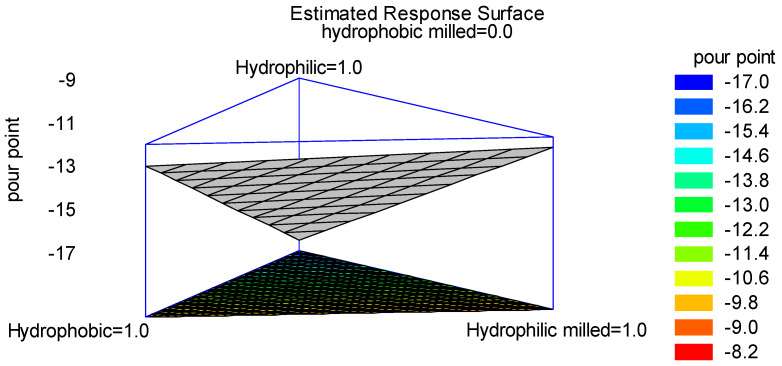
Contours of the estimated response surface.

In accordance with the simple lattice DoE [34], four experiments were conducted, and the variation in the PP, depending on the composition of the bentonite employed, was expressed in a surface plot [35], as reported in Figure 5.

Looking at the plot in Figure 5, it is possible to observe the variation in the PP in the oils treated with different bentonites. If a lower PP is desirable, hydrophilic non-milled bentonite should be used. By looking at the red and dark-orange areas on the top of the triangle, it is possible to estimate that even a mixture of hydrophilic non-milled and hydrophobic (milled or not) bentonites should guarantee a PP within the range of −15 and −17 °C.

In the end, it was highlighted how even small differences in the structure of the clay can result in a sensible variation in the ability of the material to effectively remove undesired chemicals from recycled vegetable oils. This experimental evidence is open to a wide range of customization possibilities if mixtures of different clays are considered.

## 4. Conclusions

In this study, we investigated the effectiveness of four bentonites—two commercial types (hydrophilic and hydrophobic) and two modified via 60 min of ball-milling—in processing a refined mixture of triglycerides from waste sources. The chemical structures of these bentonite powders were thoroughly characterized using FT-IR and solid-state ^29^Si and ^27^Al NMR spectroscopies.

The ^29^Si solid-state NMR results provided additional insights, particularly in the form of an increased Q4/Q3 ratio, highlighting the structural variations between the hydrophilic (S001) and hydrophobic (S002) bentonites. Furthermore, the ^27^Al solid-state NMR spectra revealed significantly lower intensity in the Al^IV^ signal in hydrophilic bentonite after milling (when comparing S001 with the milled sample, S001M1). These findings fill a critical gap left by prior studies using X-ray diffraction, SEM and TEM, which could not capture consistent differences in the chemical structures of the materials.

Equipped with this comprehensive structural characterization, the bentonites were employed as adsorbents in the treatment of recycled vegetable oil. Gas chromatography of the volatile fractions showed a higher affinity of the hydrophobic bentonite S001 for trapping ketones, along with a superior capacity to retain ketones and alcohols compared to its milled counterpart, S001M1. Additionally, the pour point (PP) was used as a further indicator of the bentonites’ ability to retain impurities during oil treatment. A simplex lattice design of experiments, combined with multivariate analysis, was applied to develop a predictive model capable of estimating the PP of treated vegetable oil based on the specific bentonite employed.

Overall, this study underscores the critical role of bentonite morphology and chemical structure, as characterized by solid-state NMR techniques, in determining their efficiency in trapping volatile organic compounds, which is related to the acidic characteristics of the material. Solid-state NMR, in particular, proved invaluable in detecting subtle yet significant differences in the structural features of bentonites, offering insights that could guide the optimization of clays for industrial applications involving VOC adsorption.

## Figures and Tables

**Table 1:** ^29^Si SS NMR spectral data for samples of hydrophilic and hydrophobic bentonite (prior to and after milling). LW indicates the NMR linewidth (Hz) measured at half-height. Q4/Q3 indicates the peak integral ratio as obtained from peak deconvolution.

Spectrum	Sample	LW Q3 (Hz)	LW Q4 (Hz)	Q4/Q3 Ratio
A	hydrophilic (S001)	491	345	0.137
B	hydrophilic milled (S001M1)	489	354	0.150
C	hydrophobic (S002)	834	476	0.190
D	hydrophobic milled (S002M2)	841	531	0.186

**Table 2:** ^27^Al SS NMR spectral data for samples of hydrophilic and hydrophobic bentonite (prior to and after milling).

Bentonite	Octahedral	d (ppm)	Al Sites Fully Condensed	d (ppm)	Al Sites in Layered Bentonite	d (ppm)
S001	91.63%	0.25	0%	-	8.36%	69.32
S001M1	91.61%	0.87	4.22%	55.70	4.15%	55.97
S002	74.50%	0.10	13.76%	58.45	11.70%	68.54
S002M1	74.50%	0.10	13.76%	58.45	11.70%	68.54

**Table 3:** List of experiments conducted and relative details.

Experiment	Bentonite	Response 1	Response 2
1	S002M1	PP *	VC **
2	S002	PP *	VC **
3	S001M1	PP *	VC **
4	S001	PP *	VC **

* PP stands for pour point; ** VC stands for volatile content.

**Table 4:** Semiquantitative volatile profiles for the triglyceride mixtures prior to bentonite treatment (TQ), and oil samples treated, respectively, with bentonites S001 (hydrophilic), S001M1 (hydrophilic milled), S002 (hydrophobic) and S002M1 (hydrophobic milled).

Compound	Retention Times (RTs) *	TQ	S001	S001M1	S002	S002M1
Pentanal	3.87	1.09	0.87	1.27	0.30	0.75
Hexanal	6.63	12.02	9.32	17.82	9.22	6.55
2-Hexenal, (E)-	10.68	0.43	0.33	0.51	0.06	0.26
Heptanal	9.69	1.21	0.85	1.80	0.04	0.58
Octanal	12.81	3.10	2.48	3.59	1.15	1.14
2-Heptenal, (Z)-	13.82	9.54	8.37	10.00	5.94	5.94
2-Octenal, (E)-	16.68	4.13	3.69	3.82	2.85	2.83
Nonanal	15.77	8.37	8.05	7.17	5.60	4.30
2-Nonenal, (E)-	19.36	1.08	1.25	0.93	1.33	1.24
2-Decenal, (Z)-	22.00	5.73	9.22	3.82	8.59	8.92
2,4-Decadienal, (E,E)-	25.74	3.23	7.92	2.90	8.69	10.14
2-Undecenal	24.44	3.23	5.42	2.05	6.41	6.97
2-Heptanone	9.61	1.36	0.53	2.00	0.12	0.33
2-Octanone	12.68	1.10	0.65	1.35	0.37	0.25
Total ketones		2.46	1.19	3.35	0.48	0.59
Acetic acid	17.83	4.00	7.31	6.34	9.67	10.59
Butanoic acid	22.34	2.12	1.41	2.09	2.61	1.94
Pentanoic acid	24.79	4.38	5.19	3.58	5.37	5.42
Hexanoic acid	27.12	15.25	11.35	11.30	15.45	16.01
Heptanoic acid	30.06	1.07	0.98	0.55	1.71	2.15
Octanoic acid	32.21	0.60	0.95	0.28	1.61	1.70
Nonanoic acid	33.81	0.32	0.59	0.13	1.24	0.94
1-Pentanol	11.86	1.17	0.83	1.32	0.84	0.61
1-Hexanol	14.80	4.12	2.03	5.73	1.89	1.73
1-Octen-3-ol	17.40	3.17	2.06	2.88	0.96	0.91
1-Heptanol	17.52	1.28	0.85	1.58	0.60	0.72
2-Hepten-1-ol, (E)-	18.94	1.03	0.60	1.10	0.68	0.48
1-Octanol	20.06	3.27	4.92	1.17	5.72	6.28
Dodecane	10.02	0.99	0.67	0.65	0.19	0.14

* Retention times are expressed in minutes.

**Table 5:** Semiquantitative assessment of the main composition of samples TQ (prior to bentonite treatment), S001 (hydrophilic bentonite), S001M1 (milled hydrophilic bentonite), S002 (hydrophobic bentonite) and S002M1 (milled hydrophobic bentonite) and their PP values.

		1	2	3	4	5
Volatiles		TQ	S001	S001M1	S002	S002M1
	Aldehydes	53.15	57.78	55.71	50.17	49.62
	Ketones	2.46	1.19	3.35	0.48	0.59
	Acids	27.73	27.77	24.26	37.64	38.76
	Alcohols	14.04	11.30	13.79	10.68	10.74
	Alkanes	0.99	0.67	0.65	0.19	0.14
Pour Point (PP, °C)		−2 °C	−16.5	−9.5	−10	−10

## Data Availability

The original contributions presented in the study are included in the article/Supplementary Material; further inquiries can be directed to the corresponding author.

## Supplementary Materials

The following supporting information can be downloaded at: https://www.mdpi.com/article/10.3390/ma18051059/s1, Figure S1: Deconvolutions of ^27^Al MAS NMR spectrum for hydrophilic bentonite; Figure S2: Deconvolutions of ^27^Al MAS NMR spectrum for hydrophilic milled bentonite; Figure S3: Deconvolutions of ^27^Al MAS NMR spectrum for hydrophobic bentonite; Figure S4: Deconvolutions of ^27^Al MAS NMR spectrum for hydrophobic milled bentonite; Figure S5: ^29^Si MAS NMR spectra of (a) hydrophilic bentonite S001, (b) ball milled hydrophilic S001M1, (c) hydrophobic bentonite S002, (d) ball milled hydrophobic bentonite S002M1; Figure S6: ^27^Al MAS NMR spectra of (a) hydrophilic bentonite S001, (b) ball milled hydrophilic bentonite S001M1, (c) hydrophobic bentonite S002, (d) ball milled hydrophobic bentonite S002M1. Spinning sidebands are marked.

## References

[1] Cárdenas J., Orjuela A., Sanchez D.L., Narváez P.C., Katryniok B., Clark J. (2021). Pre-treatment of used cooking oils for the production of green chemicals: A review. J. Clean. Prod..

[2] Foo W.H., Koay S.S.N., Chia S.R., Chia W.Y., Tang D.Y.Y., Nomanbhay S., Chew K.W. (2022). Recent advances in the conversion of waste cooking oil into value-added products: A review. Fuel.

[3] Mannu A., Garroni S., Ibanez Porras J., Mele A. (2020). Available technologies and materials for waste cooking oil recycling. Processes.

[4] Gharby S. (2022). Refining Vegetable Oils: Chemical and Physical Refining. Sci. World J..

[5] Mannu A., Almendras Flores P., Briatico F., Di Pietro M.E., Mele A. (2025). Sustainable Production of Raw Materials from Waste Cooking Oils. RSC Sustain..

[6] Mannu A., Poddighe M., Garroni S. (2022). Application of IR and UV–VIS spectroscopies and multivariate analysis for the classification of waste vegetable oils. Resour. Conserv. Recycl..

[7] Tu D., Li H., Wu Z., Zhao B., Li Y. (2014). Application of Headspace Solid-Phase Microextraction and Multivariate Analysis for the Differentiation Between Edible Oils and Waste Cooking Oil. Food Anal. Methods.

[8] Shi B., Guo X., Liu H., Jiang K., Liu L., Yan N., Farag M.A., Liu L. (2024). Dissecting Maillard reaction production in fried foods: Formation mechanisms, sensory characteristic attribution, control strategy, and gut homeostasis regulation. Food Chem..

[9] Bai S., You L., Ji C., Zhang T., Wang Y., Geng D., Gao S., Bi Y., Luo R. (2022). Formation of volatile flavor compounds, maillard reaction products and potentially hazard substance in China stir-frying beef sao zi. Food Res. Int..

[10] Lee Kuek S., Tarmizi A.H.A., Razak R.A.A., Jinap S., Norliza S., Sanny M. (2020). Contribution of lipid towards acrylamide formation during intermittent frying of French fries. Food Control..

[11] Mannu A., Di Pietro M.E., Petretto G.L., Taleb Z., Serouri A., Taleb S., Sacchetti A., Mele A. (2023). Recycling of used vegetable oils by powder adsorption. Waste Manag. Res..

[12] Mistry N., Patel V., Parekh P., Naik P., Kumar N.S., Vekariya R., Khimani M. (2024). Adsorbent Materials, Materials from Natural Sources.

[13] Mannu A., Poddighe M., Mureddu M., Castia S., Mulas G., Murgia F., Di Pietro M.E., Mele A., Garroni S. (2024). Impact of morphology of hydrophilic and hydrophobic bentonites on improving the pour point in the recycling of waste cooking oils. Appl. Clay Sci..

[14] Meirawaty M., Palit C., Setyorini D.A., Jambak M.A. (2021). Bentonite applications in simple purification of bulk cooking oil as alternative solutions for household cost efficiency. J. Community Based Environ. Eng. Manag..

[15] Aritonang B., Ritonga A.H., Harefa K., Wiratma D.Y., Herlina (2024). Purification of used Cooking Oil using a Combination of Activated Carbon and Bentonite Adsorbents. J. Farm. (JFM).

[16] (2022). Standard Test Method for Pour Point of Petroleum Products.

[17] Van Del Dool H., Kartz P.D. (1963). A generalization of the retention index system including linear temperature programmed gas-liquid partition chromatography. J. Chrom. A.

[18] Harris R.K., Becker E.D., de Menezes S.M.C., Goodfellow R., Granger P. (2001). NMR Nomenclature. Nuclear Spin Properties and Conventions for Chemical Shifts—(IUPAC Recommendations 2001). Pure Appl. Chem..

[19] Murray H.H. (2006). Chapter 6 Bentonite Applications. Dev. Clay Sci..

[20] https://www.sigmaaldrich.com/IT/it/product/sigald/285234.

[21] Hayati-Ashtiani M. (2012). Use of FTIR Spectroscopy in the Characterization of Natural and Treated Nanostructured Bentonites (Montmorillonites). Part. Sci. Technol. Int. J..

[22] Pavón E., Alba M.D. (2021). Swelling layered minerals applications: A solid state NMR overview. Prog. Nucl. Magn. Reson. Spectrosc..

[23] Lippmaa E., Magi M., Samoson A., Engelhardt G., Grimmer A.-R. (1980). Structural studies of silicates by solid-state high-resolution 29Si NMR. J. Am. Chem. Soc..

[24] Rhee C.H., Kim H.K., Chang H., Lee J.S. (2005). Nafion/sulfonated montmorillonite composite: A new concept electrolyte membrane for direct methanol fuel cells. Chem. Mater..

[25] Okada K., Arimitsu N., Kameshima Y., Nakajima A., MacKenzie K.J.D. (2006). Solid acidity of 2:1 type clay minerals activated by selective leaching. Appl. Clay Sci..

[26] Vajglová Z., Kumar N., Peurla M., Peltonen J., Heinmaad I., Yu Murzin D. (2018). Synthesis and physicochemical characterization of beta zeolite–bentonite composite materials for shaped catalysts. Catal. Sci. Technol..

[27] Danner T., Norden G., Justnes H. (2018). Characterisation of calcined raw clays suitable as supplementary cementitious materials. Appl. Clay Sci..

[28] Oloyede C.T., Jekayinfa S.O., Alade A.O., Ogunkunle O., Laseinde O.T., Adebayo A.O., Abdulkareem A.I., Smaisim G.F., Fattah I.M.R. (2023). Synthesis of Biobased Composite Heterogeneous Catalyst for Biodiesel Production Using Simplex Lattice Design Mixture: Optimization Process by Taguchi Method. Energies.

[29] Monton C., Wunnakup T., Suksaeree J., Charoenchai L., Chankana N. (2020). Investigation of the Interaction of Herbal Ingredients Contained in Triphala Recipe Using Simplex Lattice Design: Chemical Analysis Point of View. Int. J. Food Sci..

[30] Purcaro G., Szafnauer R. (2021). Tackling Food Fraud: Solid-Phase Microextraction Using Multi-Step Enrichment to Enhance Aroma Profiling in Olive Oil. Column.

[31] Choe E., Min D.B. (2007). Chemistry of Deep-Fat Frying Oils. J. Food Sci..

[32] Zhang Q., Liu C., Sun Z., Hu X., Shen Q., Wu J. (2012). Authentication of edible vegetable oils adulterated with used frying oil by Fourier Transform Infrared Spectroscopy. Food Chem..

[33] Wu C.-M., Chen S.-Y. (1992). Volatile compounds in oils after deep frying or stir frying and subsequent storage. J. Am. Oil Chem. Soc..

[34] Duquenne V. (1986). What can lattices do for experimental designs?. Math. Soc. Sci..

[35] Jankovic A., Chaudhary G., Goia F. (2021). Designing the design of experiments (DOE)—An investigation on the influence of different factorial designs on the characterization of complex systems. Energy Build..

