# Supplementary material for: Exploring the Structure–Activity Relationship of Bentonites for Enhanced Refinement of Recycled Vegetable Oil
Source: Materials (Basel). 2025 Feb 27;18(5):1059. doi: 10.3390/ma18051059 (PMC11901139; doi:10.3390/ma18051059)
Supplement: Supplementary file 1 [file materials-18-01059-s001.zip › materials-3475225-supplementary.pdf]

Exploring the Structure-Activity Relationship of Bentonites for Enhanced Refinement of Recycled Vegetable Oil

Alberto Mannu,*[1] Simona Castia,[2] Giacomo Luigi Petretto,[3] Sebastiano Garroni,[2] Franca Castiglione,[1] Andrea Mele[1]

[1] Department of Chemistry, Materials and Chemical Engineering "G. Natta", Politecnico di Milano, Piazza L. da Vinci 32, 20133 Milano, Italy; franca.castiglione@polimi.it (F.C.); andrea.mele@polimi.it (A.M.)

[2] Department of Chemical, Physics, Mathematics and Natural Science, INSTM, University of Sassari, Via Vienna 2, 07100 Sassari, Italy; a.castia@studenti.uniss.it (S.C.); sgarroni@uniss.it (S.G.)

[3] Department of Medicine, Surgery and Farmacy, University of Sassari, 07100 Sassari, Italy; gpetretto@uniss.it

* Correspondence: alberto.mannu@polimi.it

Table of Content

Figure S1	3
Figure S2	3
Figure S3	4
Figure S4	4
Figure S5	5
Figure S6	6

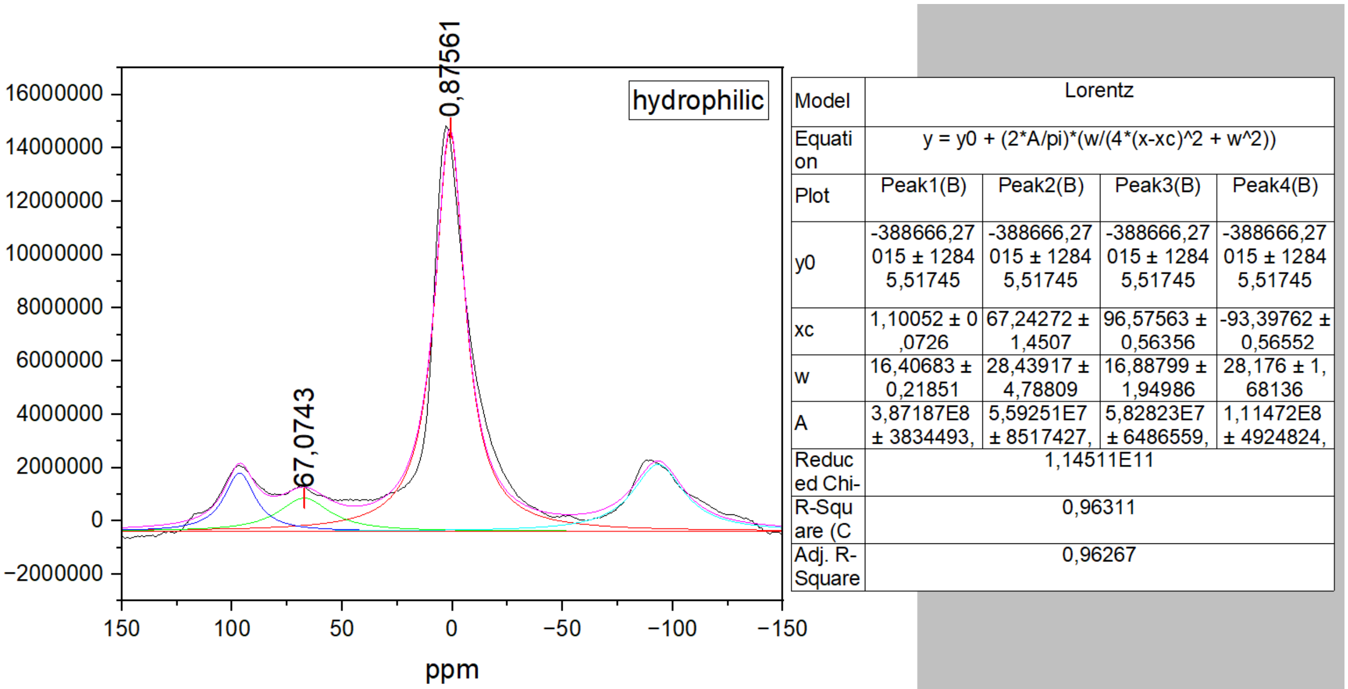

Model	Lorentz			
Equation	y = y0 + (2*A/pi)*(w/(4*(x-xc)^2 + w^2))			
Plot	Peak1(B)	Peak2(B)	Peak3(B)	Peak4(B)
y0	-388666,27015 ± 12845,51745	-388666,27015 ± 12845,51745	-388666,27015 ± 12845,51745	-388666,27015 ± 12845,51745
xc	1,10052 ± 0,0726	67,24272 ± 1,4507	96,57563 ± 0,56356	-93,39762 ± 0,56552
w	16,40683 ± 0,21851	28,43917 ± 4,78809	16,88799 ± 1,94986	28,176 ± 1,68136
A	3,87187E8 ± 3834493,	5,59251E7 ± 8517427,	5,82823E7 ± 6486559,	1,11472E8 ± 4924824,
Reduced Chi-	1,14511E11			
R-Square (C	0,96311			
Adj. R-Square	0,96267			

Figure S1. Deconvolutions of ^{27}Al MAS NMR spectrum for hydrophilic bentonite.

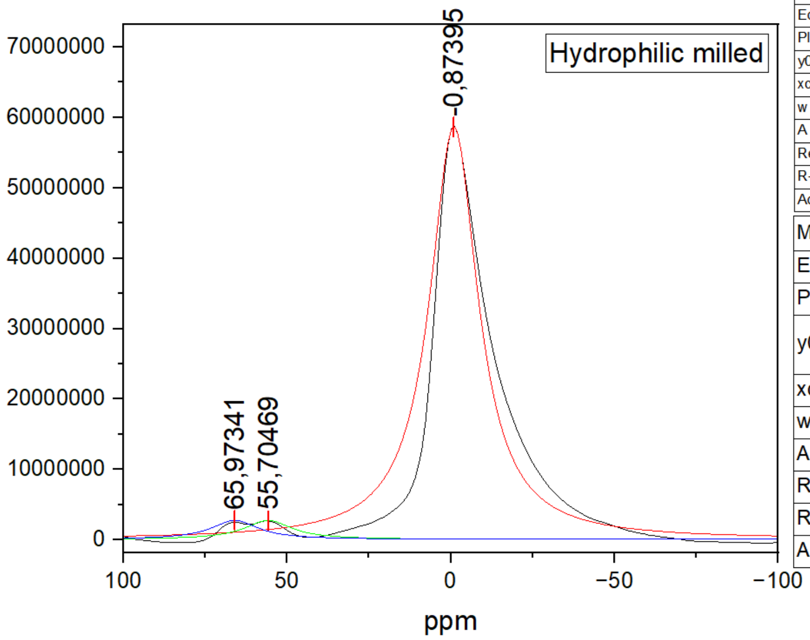

Model	Lorentz		
Equation	y = y0 + (2*A/pi)*(w/(4*(x-xc)^2 + w^2))		
Plot	Peak1(Intensity)	Peak2(Intensity)	Peak3(Intensity)
y0	96076,1875 ± 495583	96076,1875 ± 495583	96076,1875 ± 495583
xc	-0,96899 ± 0,2258	55,74555 ± 19,43825	65,94847 ± 19,36642
w	17,73371 ± 0,7606	17,73914 ± 35,66909	17,72922 ± 35,50901
A	1,63179E9 ± 5,8868E	7,45947E7 ± 3,6863E	7,45909E7 ± 3,68992
Reduced Chi-	1,54889E13		
R-Square (C	0,91831		
Adj. R-Squar	0,91454		

Model	Lorentz		
Equation	y = y0 + (2*A/pi)*(w/(4*(x-xc)^2 + w^2))		
Plot	Peak1(Intensity	Peak2(Intensity	Peak3(Intensity
y0	-759233,7085 ± 266340,9232	-759233,7085 ± 266340,9232	-759233,7085 ± 266340,9232
xc	66,09923 ± 2,2	56,49415 ± 2,1	-2,90366 ± 0,1
w	6,87812 ± 7,41	5,48697 ± 7,03	17,25699 ± 0,4
A	2,74116E7 ± 2,	2,01329E7 ± 2,	1,5975E9 ± 3,5
Reduced	6,13329E12		
R-Square	0,96765		
Adj. R-Sq	0,96616		

Figure S2. Deconvolutions of ^{27}Al MAS NMR spectrum for hydrophilic milled bentonite.

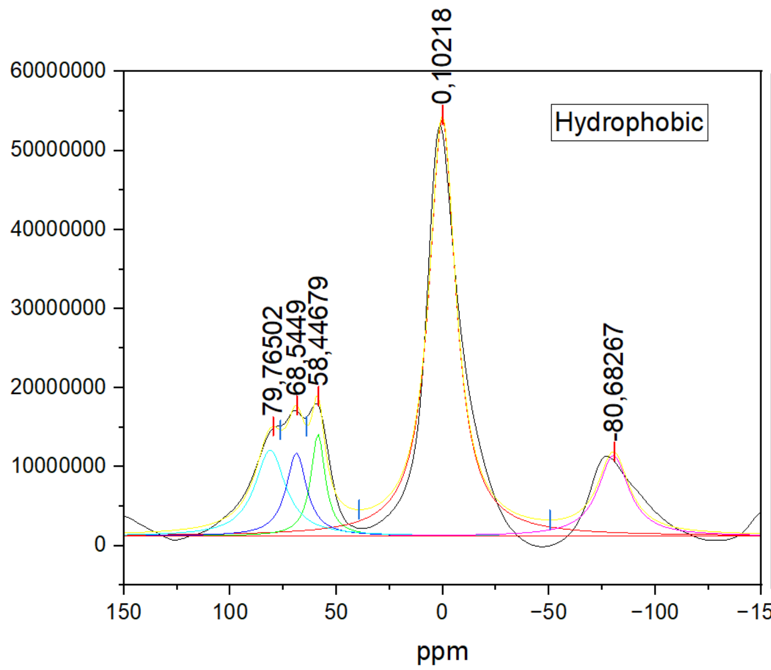

Model	Lorentz				
Equation	y = y0 + (2*A/pi)*(w/(4*(x-xc)^2 + w^2))				
Plot	Peak1(B)	Peak2(B)	Peak3(B)	Peak4(B)	Peak5(B)
y0	1230259,63541 ± 49268,34523	1230259,63541 ± 49268,34523	1230259,63541 ± 49268,34523	1230259,63541 ± 49268,34523	1230259,63541 ± 49268,34523
xc	0,1655 ± 0,07928	58,66022 ± 0,3581	68,8228 ± 0,6154	81,17861 ± 1,0224	-80,35893 ± 0,442
w	15,17344 ± 0,2314	8,09987 ± 1,2116	11,54654 ± 3,5140	17,39989 ± 2,1472	17,51661 ± 1,2908
A	1,26165E9 ± 1,395	1,64431E8 ± 3,303	1,90632E8 ± 7,750	2,97808E8 ± 5,896	2,80253E8 ± 1,503
Reduced C	1,84605E12				
R-Square (	0,95494				
Adj. R-Sq	0,95427				

Figure S3. Deconvolutions of ^{27}Al MAS NMR spectrum for hydrophobic bentonite.

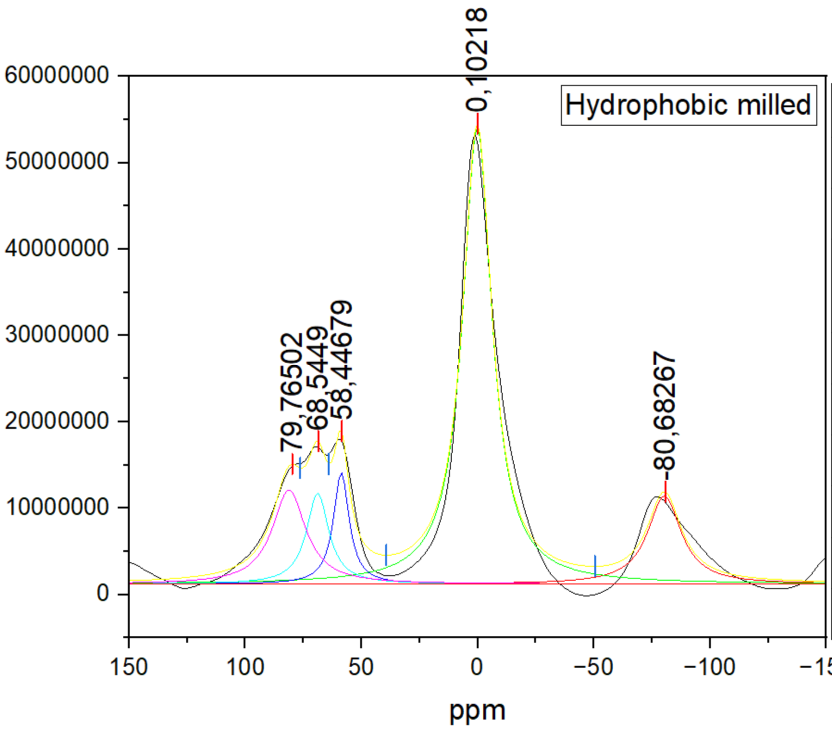

Model	Lorentz				
Equation	y = y0 + (2*A/pi)*(w/(4*(x-xc)^2 + w^2))				
Plot	Peak1(B)	Peak2(B)	Peak3(B)	Peak4(B)	Peak5(B)
y0	1230259,63649 ± 49268,34567	1230259,63649 ± 49268,34567	1230259,63649 ± 49268,34567	1230259,63649 ± 49268,34567	1230259,63649 ± 49268,34567
xc	-80,35893 ± 0,44248	0,1655 ± 0,07928	58,66022 ± 0,35818	68,8228 ± 0,61544	81,17861 ± 1,02245
w	17,51661 ± 1,29083	15,17344 ± 0,23145	8,09988 ± 1,21162	11,54653 ± 3,51401	17,39989 ± 2,14724
A	2,80253E8 ± 1,503E7	1,26165E9 ± 1,39525E7	1,64432E8 ± 3,30384E7	1,90632E8 ± 7,75014E7	2,97808E8 ± 5,89644E7
Reduced Chi-Sqr	1,84605E12				
R-Square (COD)	0,95494				
Adj. R-Square	0,95427				

Figure S4. Deconvolutions of ^{27}Al MAS NMR spectrum for hydrophobic milled bentonite.

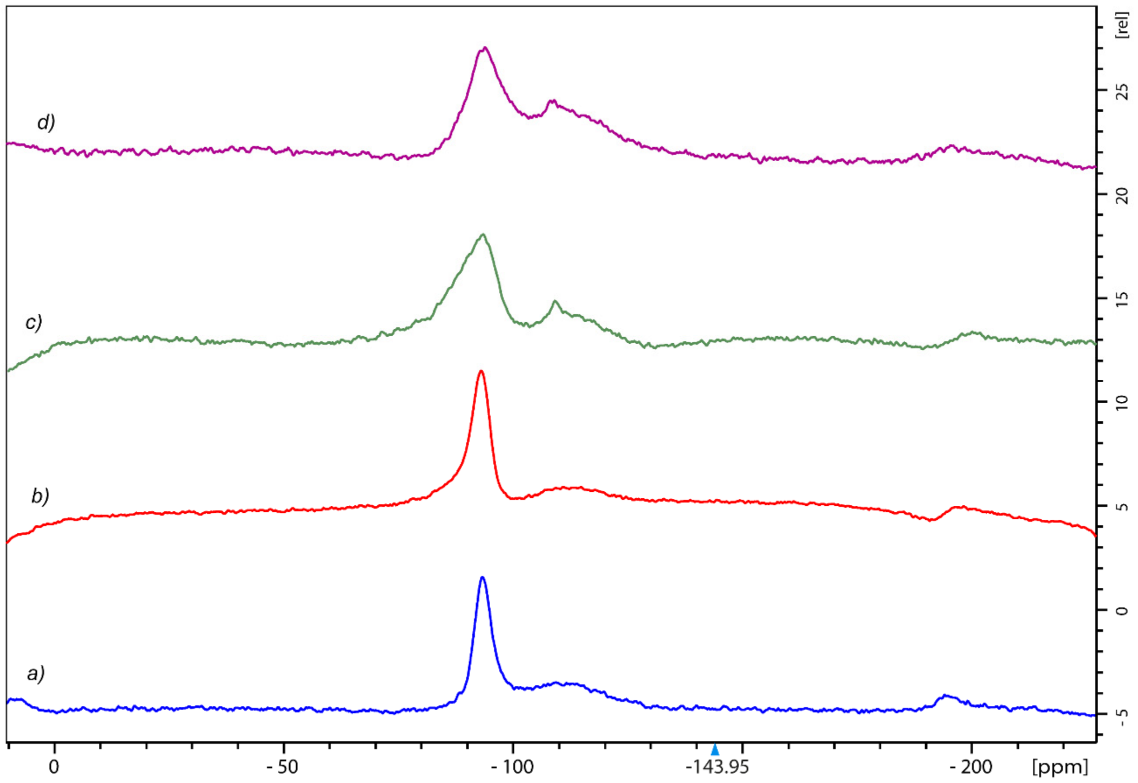

Figure S5. ^{29}Si MAS NMR spectra of a) hydrophilic bentonite S001, b) ball milled hydrophilic S001M1, c) hydrophobic bentonite S002, d) ball milled hydrophobic bentonite S002M1.

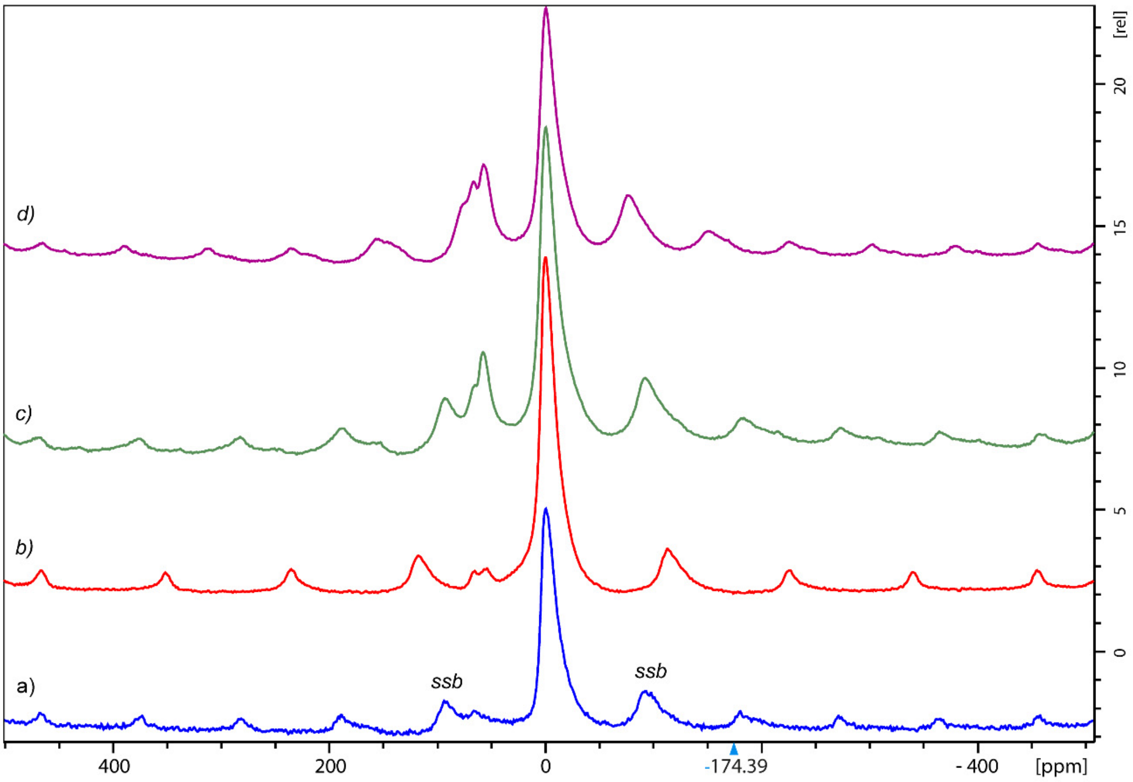

Figure S6. ^{27}Al MAS NMR spectra of a) hydrophilic bentonite S001, b) ball milled hydrophilic bentonite S001M1, c) hydrophobic bentonite S002, d) ball milled hydrophobic bentonite S002M1. Spinning sidebands are marked.
